# Continuous lighting at low PPFD improves energy efficiency while preserving growth and quality of lettuce in vertical farming systems

**DOI:** 10.3389/fpls.2026.1783548

**Published:** 2026-03-04

**Authors:** Onofrio Davide Palmitessa, Leonardo Costanza, Alessio Elia, Ettore Cantatore, Beniamino Leoni, Angelo Signore, Graziana Difonzo, Francesco Caponio, Pietro Santamaria

**Affiliations:** Department of Soil, Plant and Food Sciences, University of Bari Aldo Moro, Bari, Italy

**Keywords:** CEA, EUE, growth chamber, leafy vegetables, LUE, photoperiod

## Abstract

Vertical farming systems (VFs) offer high production efficiency in controlled environments (CEA), but their energy requirement and associated carbon footprint are strongly constrained by the high energy demand of artificial lighting is strongly constrained by the energy demand of artificial lighting. This study assessed whether different combinations of photoperiod and photosynthetic photon flux density (PPFD; 16 L:8 D at 250 µmol m⁻² s⁻¹, 12 L:12 D at 340 µmol m⁻² s⁻¹, and continuous 24 L:0 D at 170 µmol m⁻² s⁻¹) affect growth, physiology, and energy performance of two crisphead lettuce cultivars [(*Lactuca sativa* L. var. *crispa* - ‘Falstaff’ (green) and ‘Copacabana’ (red)] when the daily light integral (DLI) is maintained constant (14.4 mol m⁻² day⁻¹). Yield, morphological traits, chlorophyll fluorescence, and gas exchange parameters did not differ among lighting treatments, indicating comparable photosynthetic functioning under all photoperiod–PPFD combinations. However, continuous lighting (24 L:0 D) improved energy use efficiency (EUE) and light use efficiency (LUE), while reducing lighting costs per unit of produced biomass and demonstrating a clear benefit in terms of resource utilization. Cultivar-related differences were more pronounced than treatment effects, with red lettuce showing higher levels of phenolic compounds, carotenoids, anthocyanins, and antioxidant capacity, while maintaining similar morphological responses. Overall, the results show that under a constant DLI, photoperiod manipulation obtained by adjusting PPFD has a limited impact on plant physiology but can substantially influence yield and energy efficiency. Continuous moderate-intensity lighting thus emerges as an effective strategy to enhance the economic and environmental sustainability of VFs without compromising crop performance.

## 1 Introduction

By 2050, the global population is expected to reach 9.7 billion, with the majority living in urban areas (FAO et al., 2022). Climate change is projected to reduce crop yields by up to 60% (Rosenzweig et al., 2014), while agriculture itself contributes approximately 11% of all anthropogenic greenhouse gas emissions, positioning it as both a victim and a driver of climate change (Yan et al., 2009). In this scenario, increasing food production sustainably is one of the greatest challenges facing humanity (Wiebe et al., 2019; Malhi et al., 2021). To address this, innovative cultivation systems and advanced technologies are being adopted (Liu et al., 2022). Vertical Farming Systems (VFs) have emerged as a high-tech solution for soilless indoor cultivation, enabling the year-round production of vegetables, herbs, microgreens, and small fruits in controlled environments (van Delden et al., 2021; Ji et al., 2023). These systems, which represent a highly advanced form of protected cultivation capable of finely controlled environments, are often considered among the most promising approaches for intensive yet resource-efficient crop production. However, their overall sustainability, particularly regarding energy demand and supply-chain impacts, remains context-dependent and is debated in the literature, with some analyses highlighting significant environmental and economic trade-offs under specific conditions (Nicholson et al., 2023; Stanghellini and Katzin, 2024; Gruda et al., 2025). VFs allow precise regulation of environmental parameters, including temperature, relative humidity, CO_2_ concentration, air circulation, plant spacing, fertigation, and especially light intensity, spectrum, and photoperiod. This level of control enhances yield consistency and minimizes the variability associated with “Genotype × Environment” interactions (Kaiser et al., 2024).

Lettuce (*Lactuca sativa* L.) is one of the most widely cultivated crops in VFs due to its short growth cycle, high planting density, relatively low energy requirements, and a high edible biomass fraction, with almost the entire shoot being commercially valuable, in addition to strong market demand, particularly for minimally processed (IV gamma) products (Bantis et al., 2018; Zhang et al., 2018; Shatilov et al., 2019; Yang et al., 2022). However, maintaining stable light and climate conditions in VFs results in significantly higher energy consumption compared to open-field or greenhouse cultivation (Jin et al., 2023). Lighting alone accounts for 65–85% of total energy use, with an estimated 7–10 kWh required to produce 1 kg of lettuce (Kozai et al., 2016). Energy demand, however, varies depending on local climatic conditions, national grid efficiency, and electricity prices (Arcasi et al., 2024; Dieleman et al., 2025). For this reason, optimizing light use is critical to balancing plant productivity with energy efficiency (Arcasi et al., 2024; Stanghellini and Katzin, 2024; Hernández-Adasme et al., 2025).

Light is not only the main energy input in VFs but also a key determinant of crop quantity, quality, and morphology (Dou et al., 2018; Paradiso and Proietti, 2022);. In the fully closed systems, artificial light, especially light-emitting diode (LED), acts as the sole light source (Liu et al., 2022; Gavhane et al., 2023). Photosynthetic photon flux density (PPFD), particularly in the blue (430–453 nm) and red (642–663 nm) wavelengths, is most efficiently absorbed by chlorophylls, driving photosynthesis and related physiological processes (Hernández-Adasme et al., 2025). Other wavelengths such as ultraviolet (UV), green, and far-red (FR) can also affect plant development, influencing traits like dry weight and leaf expansion (Samuoliene et al., 2020). Defining the appropriate quantity of light is essential to avoid photoinhibition and optimize growth (Viršilė et al., 2019). The daily light integral (DLI), which combines PPFD with photoperiod, is used to determine the total PAR that a plant receives per day and is directly related to biomass accumulation, growth, and water use (Baumbauer et al., 2019; Yan et al., 2019). Nevertheless, excessively high DLI can reduce the photosynthetic quantum efficiency of PSII (Y(II)) and overall light use efficiency (LUE) (Yan et al., 2019; Matysiak et al., 2022).

For lettuce, the optimal DLI typically ranges from 12 to 16 mol·m⁻²·day⁻¹, depending on genotype (Zhang et al., 2018; Kelly et al., 2020; Modarelli et al., 2022; Boros et al., 2023;). Studies have also identified an ideal PPFD range between 150 and 300 µmol·m⁻²·s⁻¹ under artificial lighting, although higher values may also be beneficial depending on cultivar and conditions (Loconsole et al., 2019; Pennisi et al., 2020; Zhou et al., 2022). However, exceeding this range may trigger photoprotective responses that dissipate energy as heat, reducing photosynthetic efficiency and biomass accumulation (Weaver and van Iersel, 2020).

In addition to intensity, the photoperiod of light exposure is a critical variable. Under controlled-environment conditions, extending the photoperiod can contribute to increased yield and a shorter cultivation cycle (Pennisi et al., 2020) provided that specific preconditions are met, including an optimized or constant DLI, moderate PPFD levels to prevent photoinhibition, and the use of light-tolerant species such as lettuce. In this context, yield responses are mainly driven by the redistribution of the same DLI over a longer photoperiod rather than by photoperiod extension per se ( (Koontz and Prince, 1986; Gaudreau et al., 1994; Palmer and van Iersel, 2020). For instance, Chen et al. (2022) reported that butterhead lettuce responded positively to 24 hours of continuous light cycle under combined red and blue light, achieving high fresh weight without negative physiological effects (Velez-Ramirez et al., 2011).

Given that electricity consumption for artificial lighting can account for approximately 20–40% of the total operating costs in VFs, depending on electricity prices and system efficiency (Yorifuji and Obara, 2022), this study aims to identify the optimal combination of photoperiod and PPFD, maintaining the DLI at an optimal target level of 14 mol·m⁻²·day⁻¹, for two lettuce cultivars grown in a growth chamber, with a focus on improving energy use efficiency (EUE) and optimizing light management.

## 2 Material and methods

### 2.1 Plant material and growing conditions

The study was carried out in a walk-in growth chamber (3.36 × 2.86 × 2.78 m) designed to ensure precise environmental control. The chamber features sandwich panel walls with 80 mm polyurethane insulation, covered with pre-painted galvanized steel to guarantee thermal stability [K = 0.23 kcal/(h·m²·°C)]. The environmental parameters [temperature, relative humidity (RH), CO_2_ concentration, and lighting] were managed via an integrated Programmable Logic Controller -based electronic control unit with touchscreen interface and remote web access. The system allowed the creation of up to eight programmable daily time slots for each parameter, with real-time and historical data monitoring. Lighting was provided by dimmable LED lamps (3.2–3.4 µmol·J⁻¹), with customized spectra for each shelf level and a total photon flux of 900–1100 µmol·s⁻¹ for the ceiling-mounted units (Aquila series, Ambralight, Italy). The growth chamber hosted three movable aluminum racks (each 60 × 189 × 200 cm) equipped with hydroponic aluminum trays and independent irrigation systems, including stainless-steel centrifugal pumps, mesh filters, and nutrient solution tanks (200 L each). Temperature and RH were regulated by an air-cooled condensing unit and a dual-air evaporator with stainless-steel heating resistors. Air recirculation, de-stratification, and automated humidification ensured homogeneity throughout the growing space. The CO_2_ injection system included an electrovalve, pressure reducer, and monitoring sensors. All climatic and technical parameters were continuously monitored and recorded, ensuring standardized and reproducible growth conditions. Two cultivars (cv) of crisphead lettuce (*Lactuca sativa* L. var. *crispa*) - ‘Falstaff’ (green) and ‘Copacabana’ (red) (Isi Sementi) - were grown in two crop cycles.

The seeds were sown in rockwool plugs (Grodan plantop plug NG2.0, Roermond, The Netherlands) in polystyrene trays (600 × 400 × 50 mm) with 240 holes (1,000 seedlings·m^-2^). At the stage of two true leaves, the seedlings were transplanted into 0.5 L pots filled with a 1:1 (v/v) mixture of fine and medium-grade perlite (Perlite Italiana, Italy). The fine-grade perlite had a particle size of 0.5–1.5 mm, while the medium-grade perlite ranged from 1.5–3.0 mm. On each shelf, 16 pots (eight for each of the two lettuce cultivars) were placed.

**Figure 1:**
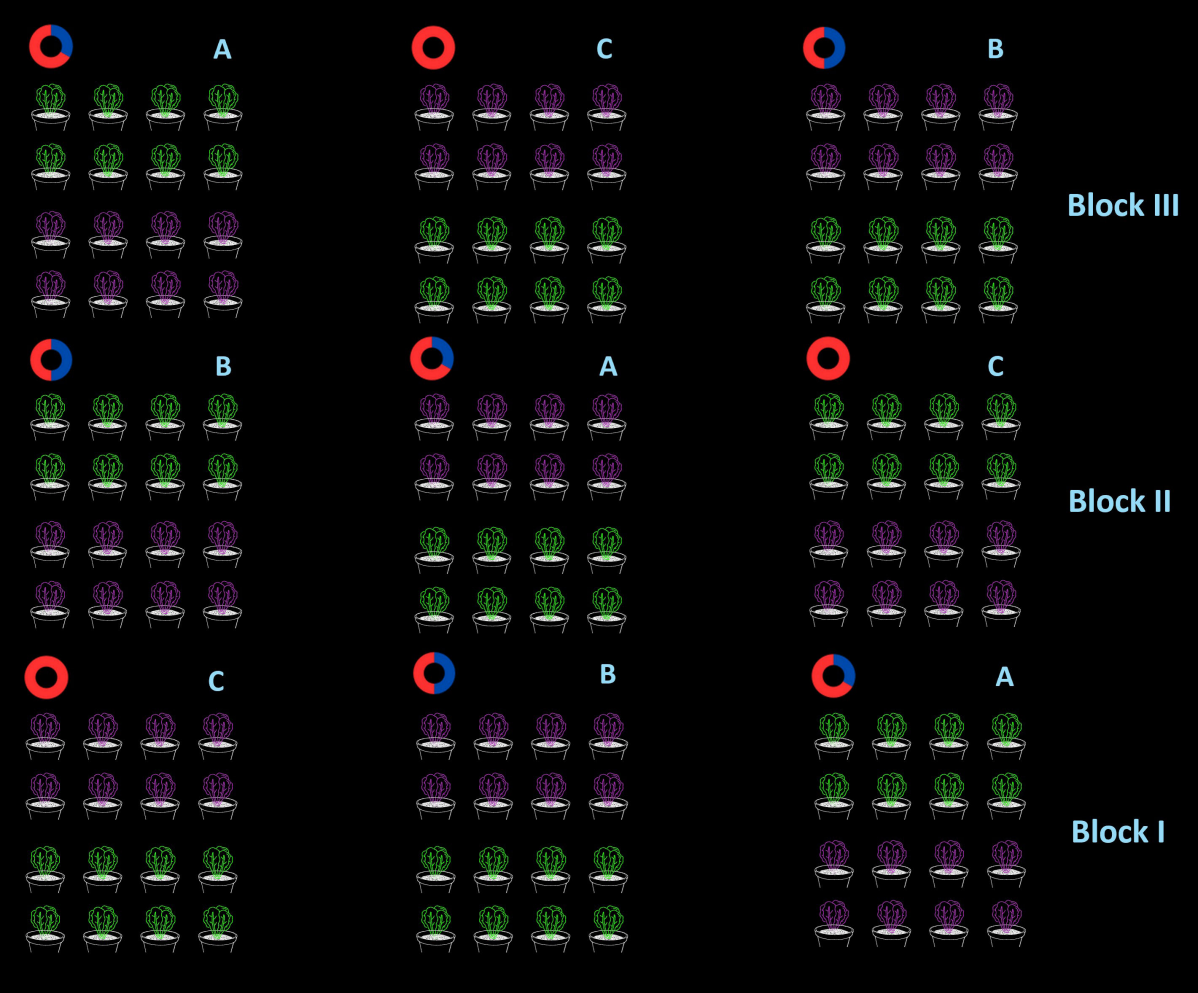
Schematic representation of the experimental design adopted in the walk-in growth chamber. Each shelf hosted 16 pots (eight per lettuce cultivar), arranged to reach a planting density of 33.3 plants·m⁻².

The pots were placed at 15 cm between the rows and 20 cm within the row, with a final shelf density of 33.3 plants·m^-^² (**Figure 1**). Fertigation was managed in an ebb and flow system (closed cycle), with a total of four irrigations per day, with a timing of 10 minutes (pump flow rate 20 L·min⁻¹).

The nutrient solution (NS) had the following concentrations of macro-nutrients (expressed in mmol·L⁻¹): 5.42 N-NO_3_, 0.29 N-NH_4_, 2.71 K, 0.65 P, 0.74 Mg, 1.75 Ca, 1.00 S. While the micronutrients composition, expressed as µmol·L^-1^, was: 20 Fe, 5 Mn, 2.5 Zn, 25 B, 0.75 Cu, and 0.5 Mo. These concentrations represent calculated target values, derived from the stoichiometric formulation of the nutrient solution based on the fertilizers used. During the crop cycles, the electrical conductivity (EC) and pH of the NS were maintained within a range of 1.2-2.0 dS·m⁻¹ and 5.5-6.5, respectively. During the germination phase, the growth chamber was kept with a temperature of 20 °C, RH of 80%. The CO_2_ concentration inside the growth chamber was at ambient atmospheric levels (≈400 µmol·mol⁻¹). Nevertheless, after transplanting, air temperature and relative humidity were set to 24 °C and 65%, respectively, as these conditions are widely reported as optimal for lettuce cultivation under controlled-environment agriculture. This temperature range supports high photosynthetic activity and biomass accumulation while avoiding heat stress, whereas a relative humidity around 60–70% ensures adequate transpiration and stomatal conductance without increasing the risk of physiological disorders or microbial development. These settings are commonly adopted in growth chamber and vertical farming studies to standardize environmental conditions and minimize confounding effects unrelated to lighting treatments (Pennisi et al., 2020).

### 2.2 Light treatments

**Figure 2:**
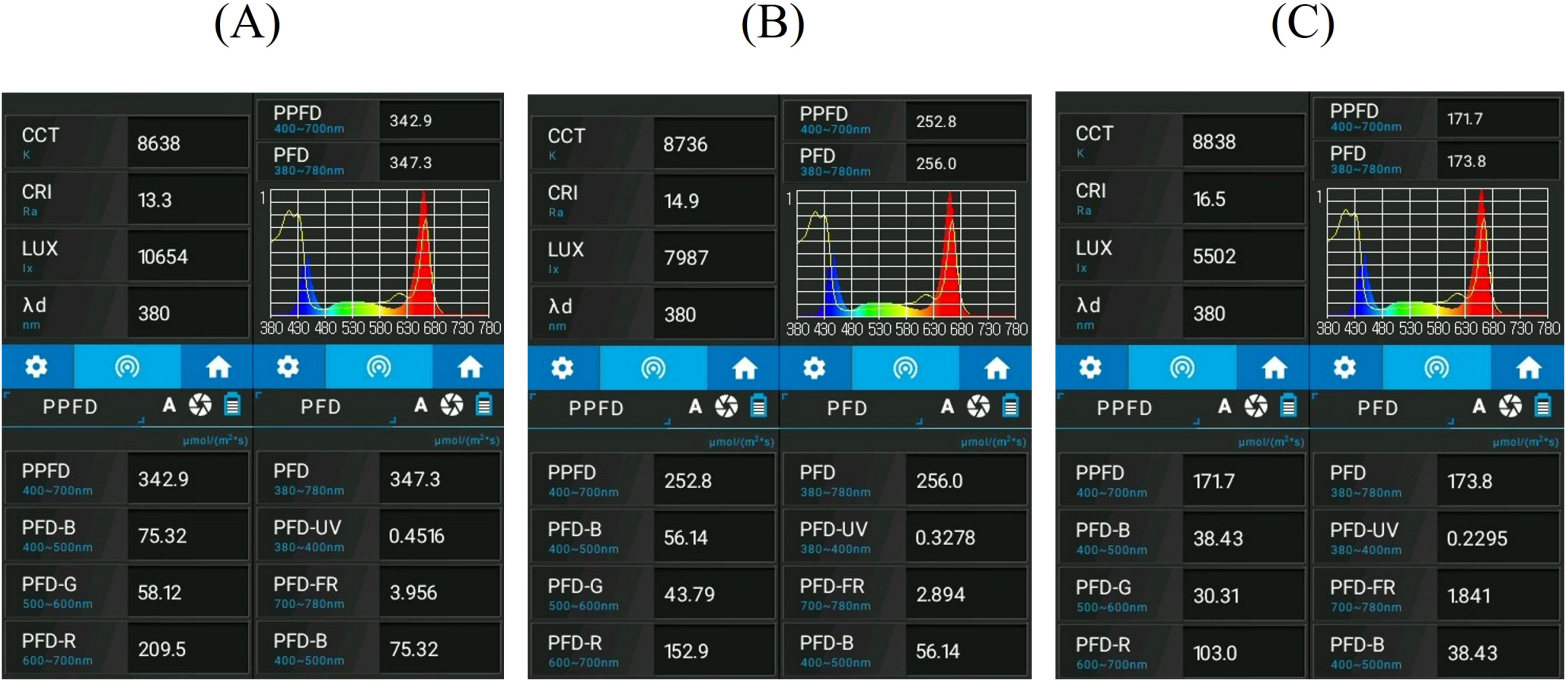
Spectral and photometric parameters measured under three different lighting treatments using a portable spectroradiometer. Panels show values of correlated color temperature (CCT), color rendering index (CRI), illuminance (LUX), dominant wavelength. **(A–C)** correspond to light treatment 16 L:8 D, 12 L:12 D and 24 L:0 D respectively.

LED lamps were used, with a “full light spectrum” (**Figure 2**), a photosynthetic photon flux (PPF) of 80–200 µmol s⁻¹ and efficiency ranging from 3.0 to 3.4 µmol J⁻¹. Each unit (length:120 cm, weight:680 g) operates at 25–60 W and is rated IP65, with integrated protections against short circuit, overvoltage, and overheating. The emission spectrum of the LED lamps was measured *in situ* using a LI-180 Spectroradiometer (LI-COR Biosciences, Lincoln, NE, USA). PPFD and spectral distribution were measured at a fixed reference height corresponding to the average canopy level of lettuce plants during the main vegetative phase, rather than directly at the tray surface, using a portable spectroradiometer (LI-180, LI-COR Biosciences, Lincoln, NE, USA), operating in the 380–780 nm range, with a spectral resolution of 1 nm and a cosine-corrected sensor. PPFD measurements were collected at multiple points across each shelf and are reported as mean ± standard deviation. The instrument provides PPFD measurements with an accuracy of ± 5% and was factory calibrated prior to use. PPFD measurements were performed at leaf level to characterize the actual light quality received by the plants under each lighting treatment, following a grid-based approach, with multiple points evenly distributed across each shelf to account for spatial variability. Reported PPFD values represent the mean ± standard deviation of these measurements. Spectral data are expressed as PPFD (μmol·m⁻²·s⁻¹; **Figure 2**). Each shelf (or light treatment) was isolated using blackout curtains to prevent light contamination between treatments. Photoperiod and PPFD treatments were set as follows:

- photoperiod 16:8 h (light/dark), with PPFD equal to 250 ± 10 μmol·m⁻²·s⁻¹ (16 L:8 D).
- photoperiod 12:12 h (light/dark), with PPFD equal to 340 ± 12 μmol·m⁻²·s⁻¹ (12 L:12 D).
- photoperiod 24:0 h (light/dark), with PPFD equal to 170 ± 8 μmol·m⁻²·s⁻¹ (24 L:0 D).

All treatments were set to achieve the same DLI value of 14.4 ± 0.4 mol·m⁻²·day⁻¹.

### 2.3 Harvesting analysis

At harvest time (26 days after transplant; DAT) Destructive measurements were performed on four plants per experimental unit to evaluate key morphological traits. Specifically, the length and width of the largest fully expanded leaf, leaf area (LA), shoot fresh weight, and shoot dry weight were recorded. Shoot fresh weight was measured immediately after harvest using a precision electronic balance, while shoot dry weight was obtained by drying leaves in a ventilated oven at 80 °C until a constant weight was reached. LA was measured using a LI-3100C Area Meter (LI-COR Biosciences, Lincoln, NE, USA). Leaves were manually arranged flat on the illuminated reading surface and scanned with a high-resolution optical system capable of accurately detecting the two-dimensional profile of the leaf blade. This method enabled rapid and reproducible LA estimation, minimizing operator-related bias even in leaves with irregular margins. Colorimetric measurements were conducted with a CM-700d handheld spectrophotometer (Konica Minolta, Tokyo, Japan), featuring a wavelength range of 400–700 nm, spectral interval of 10 nm, and repeatability of ΔE*ab ≤ 0.04 under standard conditions and operating in diffuse reflectance mode (D65 illumination, 10° observer angle), following the CIE guidelines. The instrument was calibrated with a white reference tile, and the aD65*, bD65*, L*D65, and *Chroma* values of each leaf were recorded based on the CIE Lab* system.

### 2.4 Plant physiology measurements

The day before harvest operation, 25 DAT, chlorophyll fluorescence was measured using a pulse-amplitude-modulated fluorometer (PAM-2500, Heinz Walz GmbH, Germany), equipped with a blue measuring light (λ ≈ 470 nm) and saturating pulses exceeding 6,000 µmol photons·m⁻²·s⁻¹. The device allows detection of fluorescence signals with high temporal resolution (up to 10 µs), ensuring accurate determination of PSII photochemical parameters. The measurements were made at 25 DAT, including: i) Fv/Fm: the maximum quantum efficiency of Photosystem II (PSII), calculated after dark adaptation (it represents the potential efficiency of PSII photochemistry when all reaction centers are open; healthy, non-stressed plants typically show values around 0.83; a lower Fv/Fm indicates stress or photoinhibition; ii) ETR (Electron Transport Rate): the rate of electron flow through PSII during photosynthesis (it is an indicator of photosynthetic); iii) Y(II): the effective quantum yield of PSII in light-adapted conditions (it reflects the proportion of light absorbed by chlorophyll that is used in photochemistry under real conditions); iv) NPQ (Non-Photochemical Quenching): a measure of non-photochemical energy dissipation (it reflects how much excess light energy is being dissipated as heat to protect the photosynthetic apparatus, especially under high light stress).

Before measurements, leaves were kept in the dark for 30 minutes using a dark clip, allowing a full re-open of all PSII reaction centers. This dark adaptation ensures accurate detection of i) Fo: the minimal fluorescence when all PSII reaction centers are open (no photochemical activity); ii) Fm: the maximal fluorescence when all PSII centers are closed after a saturating light pulse.

In addition to these steady-state parameters, at 25 DAT, fast chlorophyll fluorescence kinetics were recorded to analyze the rapid changes of fluorescence occurring in milliseconds after illumination. Rapid light curves were performed with the PAM-2500 with gradually increasing irradiance in seven steps. For each step, the irradiance was 40, 140, 270, 500, 870, 1400, and 2000 μmol·m^-2^·s^-1^, and the fluorescence signal was recorded. Data were recorded using the software by PamWin-4 V4.02v. In addition, the maximum quantum yield for whole chain electron transport, at low light intensities (*Alpha*), the maximum electron transport capacity, at light saturation (*ETRmax*) and the light saturation coefficient (*Ik*), was taken. Fluorescence was simultaneously detected at wavelengths above 700 nm and below 710 nm using dual detectors to differentiate between PSII and PSI signals. This dual-emission analysis enables a more complete understanding of the interplay between the two photosystems and how plants respond to light stress or physiological changes.

Furthermore, gas exchange parameters, including net assimilation rate (*An*), stomatal conductance (*gs*), and transpiration rate (*E*), were performed at 25 DAT, using a portable photosynthesis system (LI-6400XT, LI-COR Biosciences, Lincoln, NE, USA), equipped with a 2 × 3 cm clear-top leaf chamber and an infrared gas analyzer with a CO_2_ measurement accuracy of ±1 µmol·mol⁻¹ and H_2_O accuracy of ±0.1 mmol·mol⁻¹. The system was configured with a CO_2_ concentration of 400 ppm, a leaf chamber temperature of 25 °C, and an airflow rate of 500 μmol·s⁻¹, ensuring stable and controlled environmental conditions. For the plant physiology parameters, two plants per experimental unit were measured, and one representative, fully expanded leaf was used per plant.

### 2.5 Energy use efficiency

LED-related electricity consumption (considering different lighting conditions as described in **Figure 1**) was monitored using energy meters. In the same way, the consumption of other system components (HVAC system and cultivation facilities, e.g., water pumps and fertigation units) was monitored for the entire growth period. The Energy use efficiency (EUE), expressed as g·kWh^-1^, was calculated as follow (Equation 1); Plant fresh weight (PFW) was expressed as g·plant⁻¹ and measured at harvest; plant density is 33.3 plants·m^-^², and LED power consumption is the electricity consumption per square meter of surface of artificial light treatment, obtained by multiplying the power consumption of the LEDs over their operating time (kWh).):

(1) $$EUE=\frac{PFW\cdot Plant\;density}{LED\;power\;consumption}$$

The calculation of LEDs electrical energy cost, expressed as €·m^-2^, was made by multiplying the hourly energy consumption per square meter of surface of LEDs system for each treatment by the corresponding hourly tariff rates, as obtained from the Italian electricity market data (https://www.mercatoelettrico.org/It/Statistiche/ME/DatiSintesi.aspx - accessed on 07/07/2025) Equation 2:

Finally, light use efficiency (LUE) was evaluated (Equation 2). It describes how effectively the plants converted light into biomass and indicates how much plant growth results from the light absorbed. It is expressed as g · mol^-1,^ and it was calculated with the following formula (2):

(2) $$LUE=\frac{PFW\cdot Plant\;density}{DLI}$$

where the DLI is obtained by multiplying the PPFD of the LEDs by the duration of their operation and converting the result into moles of photons per square meter per day. These parameters provided quantitative benchmarks for optimizing energy input, guiding the adoption of energy-saving technologies and light management strategies to enhance sustainability and cost-effectiveness in indoor cultivation.

### 2.6 Biochemical analyses

To investigate the effects of light treatments on the biochemical content of lettuce, biochemical analyses were performed on freeze-dried leaf samples. Total chlorophyll (a + b) and carotenoids were quantified by spectrophotometric analysis in acetone extracts, according to Lichtenthaler and Buschmann (2001). Absorbance was recorded at 661.6, 644.8, and 470 nm, and pigment concentrations were calculated using established equations. Total phenolic content (TPC) and antioxidant activity (ABTS assay) were determined following Admane et al. (2023) and Tarantino et al. (2020), using ethanolic extracts of freeze-dried leaves. TPC was expressed as mg gallic acid equivalents (GAE) per g of dry weight, while antioxidant capacity was evaluated by ABTS (2,2’-azino-bis(3-ethylbenzothiazoline-6-sulfonic acid) assay and expressed as µmol Trolox equivalents (TE) per g of dry weight. Total anthocyanin content (TAC) was measured only in GCII by UV–vis spectrophotometry, using extracts obtained from the **entire aboveground biomass of each plant**, following extraction with methanol–water–formic acid (50:44:6, v/v/v). Absorbance at 535 nm was used to quantify TAC, expressed as mg malvidin-3-O-glucoside per g of dry weight. Absorbance measurements were performed using a UV–Vis spectrophotometer with a wavelength accuracy of ±1 nm and photometric accuracy of ±0.005 absorbance units, ensuring reliable quantification of pigments and antioxidant-related compounds.

### 2.7 Statistical analysis

Experimental design was a split-plot scheme, with light treatments representing the main plots and cultivars the sub-plots (**Figure 1**). Statistical analysis was conducted using R software (version 4.4.2. - R Core Team (2024)) within the RStudio environment (RStudio for Windows, Version 2025.05.0 + 496, Posit Software, PBC, Boston, MA, USA) (Posit Team 2025). To assess differences between experimental treatments, a two-way analysis of variance (ANOVA) was applied, after checking the assumptions of normality and homoscedasticity by the Shapiro-Wilk and Levene test, respectively. In the presence of significant effects (p< 0.05), multiple comparisons between the means, Tukey HSD *post-hoc* test was implemented.

Despite having conducted two experimental trials, the results presented here referred only to the second trial, which was performed in the growth chamber under strictly controlled and consistent environmental and cultivation conditions. This approach ensures data reliability and minimizes variability not caused by experimental factors.

## 3 Results

### 3.1 Lettuce morphology and production

The growth cycle of the two lettuces cultivated in the growth chamber with three light treatments (12 L:12 D, 16 L:8 D, and 24 L:0 D) was 26 DAT. Light treatments did not influence plant morphology: on average, number, length and width of leaves per plant and plant fresh weight (PFW) were 14.6, 161.4 mm, 106.4 mm and 39.5 g·plant^-1^, respectively (**Table 1**). Independently to light treatments, plants of green lettuce had 2.6 leaves more than red ones, and PFW was 18% more in green than red lettuce (**Table 1**). Differently, the leaves of red lettuce were 43.2% longer compared to those of green one (**Table 1**).

**Table 1:** Morphological traits of two lettuce cultivars, ‘Falstaff’ (green) and ‘Copacabana’ (red), grown under three photoperiods and with the same DLI. .

	Leaf number	Leaf length	Leaf width	Plant fresh weight
	n./plant	mm	mm	g/plant
Photoperiod (P)				
16 L:8 D	13.8 ± 1.24	160.7 ± 16.3	104.6 ± 6.0	38.5 ± 1.8
12 L:12 D	14.9 ± 1.13	163.4 ± 13.9	105.3 ± 4.7	38.0 ± 1.8
24 L:0 D	15.0 ± 1.00	160.0 ± 15.7	109.4 ± 5.5	42.0 ± 2.2
Cultivar (Cv)				
Green	15.8 ± 1.1	139.8 ± 9.0	101.5 ± 6.8	42.9 ± 3.4
Red	13.2 ± 1.0	183.0 ± 11.5	111.4 ± 9.2	36.1 ± 3.2
Significance^(1)^				
P	ns	ns	ns	ns
Cv	*	**	ns	*
P x Cv	ns	ns	ns	ns

Mean ± SE values are reported.

^(1)^ Significance: ** and * for p ≤ 0.01 and p ≤ 0.05, respectively; ns, not significant. Different lowercase letters indicate significant differences (p = 0.05) between means within the same column (Tukey HSD).

Furthermore, the treatments did not influence leaf area, specific leaf area and yield (*p* = 0.08) with the average values of 805 cm^2^, 115.2 cm²·g^-1^ and 1.3 kg·m^-2^ respectively (**Table 2**). Between the cultivars, red lettuce showed 9% more dry matter content than green one but green lettuce produced 222 g·m^-2^ more than red one (**Table 2**).

**Table 2:** Dry matter content, leaf area, specific leaf area, and yield of two lettuce cultivars, ‘Falstaff’ (green) and ‘Copacabana’ (red), grown under three photoperiods and with the same DLI.

	Dry matter	Leaf area	Specific leaf area	Yield
	g·100 g^-1^ of fresh weight	cm²·plant^-1^	cm²·g^-1^	g·m^-2^
Photoperiod (P)				
16 L:8 D	7.1 ± 0.31	818.8 ± 89.2	115.6 ± 13.3	1271 ± 44.2 b
12 L:12 D	6.9 ± 0.34	799.8 ± 85.7	117.0 ± 15.4	1255 ± 43.9 b
24 L:0 D	7.1 ± 0.38	796.7 ± 91.3	113.8 ± 14.0	1390 ± 54.9 a
Cultivar (Cv)				
Green	6.7 ± 0.36	747.9 ± 86.2	112.3 ± 16.4	1415 ± 79.6
Red	7.3 ± 0.37	862.3 ± 81.7	118.7 ± 17.9	1193 ± 70.8
Significance^(1)^				
P	ns	ns	ns	ns
Cv	*	ns	ns	**
P x Cv	ns	ns	ns	ns

Mean ± SE values are reported.

^(1)^ Significance: ** and * for p ≤ 0.01 and p ≤ 0.05, respectively; ns, not significant. Different letters indicate significant differences (p = 0.05) between means within the same column (Tukey HSD).

### 3.2 Fluorescence parameters and gas exchange system

Chlorophyll fluorescence parameters were not affected by light treatments or cultivars, with overall mean values of 0.45 for Y(II), 0.95 for NPQ, 50.0 µmol m⁻² s⁻¹ for ETR, and 0.78 for Fv/Fm (**Supplementary Table 1**).

Consistently with chlorophyll fluorescence parameters, no effects of photoperiod or cultivar were observed for the parameters derived from the light response curves. Overall mean values were 62.3 µmol·m⁻²·s⁻¹ for *ETRmax*, 0.38 for *α*, and 170.5 µmol photons·m⁻²·s⁻¹ for *Ik* (**Supplementary Table 2**).

Net assimilation rate (*An*) was not affected by light treatments and cultivars, and its average value was 37.9 µmol CO_2_·m^-2^·s^-1^ (**Table 3**). Differently, *gs* was 0.20 mol H_2_O·m^-2^·s ^-1^ more for green than for red lettuce (**Table 3**). Similarly, *E* was not affected by light treatments, but it was 1.5 times more for green lettuce than for red ones (**Table 3**).

**Table 3:** Net assimilation rate (*An*), stomatal conductance (*gs*), and transpiration rate (*E*) of two lettuce cultivars, ‘Falstaff’ (green) and ‘Copacabana’ (red), grown under three photoperiods and with the same DLI.

	An	gs	E
	µmol CO_2_·m^-2^·s^-1^	mol H_2_O·m^-2^·s^-1^	mmol H_2_O·m^-2^·s^-1^
Photoperiod (P)			
16 L:8 D	38.1 ± 1.0	0.20 ± 0.08	3.90 ± 1.38
12 L:12 D	38.1 ± 0.9	0.17 ± 0.08	3.28 ± 1.24
24 L:0 D	37.3 ± 0.7	0.21 ± 0.07	3.87 ± 0.90
Cultivar (Cv)			
Green	38.4 ± 1.3	0.30 ± 0.06	5.30 ± 0.72
Red	37.3 ± 0.7	0.10 ± 0.04	2.07 ± 0.81
Significance^(1)^			
P	ns	ns	ns
cv	ns	*	*
P x Cv	ns	ns	ns

Mean ± SD values are reported.

^(1)^ Significance: * for p ≤ 0.05; ns, not significant.

### 3.3 LEDs electrical energy consumption and cost, energy use efficiency and light use efficiency

During the growth cycle, the average LEDs electrical consumption was 130 kWh·m^-2^, independently to light treatments (**Table 4**). But the cost of LEDs application was 16.5% lower for the continuous light (24 L:0 D) compared with the other treatments (**Table 4**). Consequently, the best performances in terms of EUE and LUE were found for the lettuces grown under continuous light treatment, with EUE and LUE respectively 21% and 12% better than for the lettuces grown under 16 L:8 D and 12 L:12 D (**Table 4**). Furthermore, green lettuce showed 1.7 g·kWh^-1^ (EUE) and 0.6 g·mol^-1^ (LUE) more than red lettuce (**Table 4**).

**Table 4:** LEDs electrical consumption, LEDs energy costs, energy use efficiency (EUE), and light use efficiency (LUE) of two lettuce cultivars, ‘Falstaff’ (green) and ‘Copacabana’ (red), grown under three photoperiods and with the same DLI.

	LEDs electrical consumption	LEDs energy cost	EUE	LUE
	kW·m^-2^	€·m^-2^	g·kWh^-1^	g·mol^-1^
Photoperiod (P)				
16 L:8 D	129 ± 10.02	18.2 ± 1.53 a	9.8 ± 1.07 b	3.4 ± 0.21 b
12 L:12 D	140 ± 17.29	19.8 ± 2.44 a	9.0 ± 0.98 b	3.4 ± 0.22 b
24 L:0 D	122 ± 12.25	16.3 ± 1.79 b	11.4 ± 1.38 a	3.8 ± 0.22 a
Cultivar (Cv)				
Green	130 ± 13.2	18.4 ± 1.95	10.9 ± 1.45	3.8 ± 0.52
Red	130 ± 20.8	18.4 ± 3.01	9.2 ± 1.46	3.2 ± 0.49
Significance^(1)^				
P	ns	**	**	*
Cv	ns	ns	***	***
P x Cv	ns	ns	ns	ns

Mean ± SE values are reported. .

^(1)^ Significance: ***, ** and * for p ≤ 0.001, p ≤ 0.01 and p ≤ 0.05, respectively; ns, not significant. Different letters indicate significant differences (p = 0.05) between means within the same column (Tukey HSD).

### 3.4 Color parameters and bioactive compounds content

Light treatments did not influence the colorimetric parameters of green (**Supplementary Table 3**) and red lettuce (**Supplementary Table 4**). On average, among light treatment: aD65* was -13.3 and 2.23, respectively for green and red lettuce (**Supplementary Tables S3**, **S4**); bD65* was 19.33 and 0.97, respectively for green and red lettuce (**Supplementary Tables S3**, **S4**); L*D65* was 42.07 and 28.47, respectively for green and red lettuce (**Supplementary Tables S3**, **S4**); Chroma was 23.47 and 2.97, respectively for green and red lettuce (**Supplementary Tables S3**, **S4**).

**Figure 3:**
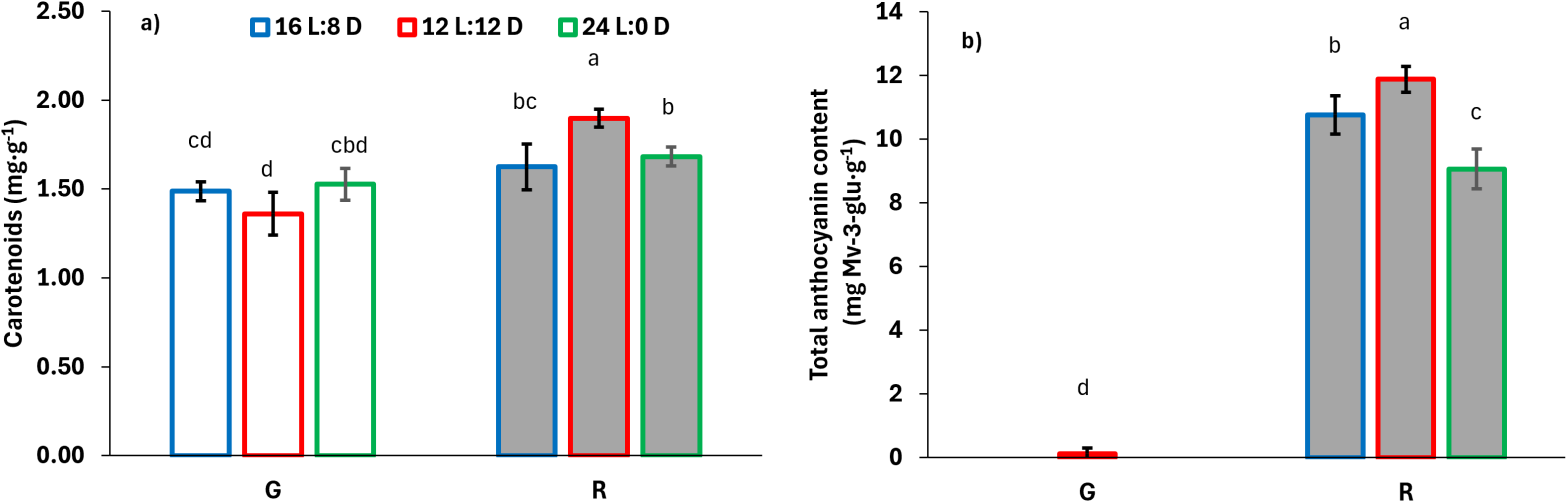
**(a)** Carotenoid content and **(b)** total anthocyanin content of two lettuce cultivars, ‘Falstaff’ (green; G) and ‘Copacabana’ (red; R) grown with three lighting treatments. On **(b)** total anthocyanin content were not detectable in green lettuce under the 16 L:8 D and 24 L:0 D treatments; therefore, bars for these combinations are not shown. Vertical bars represent ± SE. Different letters indicate significant differences between light treatments and cultivars (Tukey HSD, p = 0.05).

Similarly to color parameters, light treatments did not affect chlorophyll content or antioxidant capacity (ABTS) of lettuce (**Table 5**). However, differences between cultivar were observed: green lettuce exhibited a 22% higher chlorophyll content, whereas red lettuce showed ABTS values approximately 2.38-fold higher (**Table 5**). Carotenoid content was influenced by cultivar x photoperiod interaction. The highest carotenoid concentration was observed in red lettuce grown under 12 L:12 D (**Figure 3a**). Red lettuce grown under 16 L:8 D and 24 L:0 D still exhibited carotenoid contents 21% higher than those observed in green lettuce grown under 12 L:12 D (**Figure 3a**). On average, total phenolic content (TPC) was 11% higher under the 16 L:8 D photoperiod compared with other light treatments and was 2.65-fold higher in red lettuce compared with green lettuce (**Table 5**). Anthocyanins were not detected in green lettuce, except for a negligible amount in plants grown under 12 L:12 D (**Figure 3b**). In contrast, substantially elevated anthocyanin levels were measured in red lettuce. Red lettuce grown under 12 L:12 D showed anthocyanin contents 13% and 22% higher than those observed under 16 L:8 D and 24 L:0 D, respectively (**Figure 3b**).

**Table 5:** Chlorophylls, carotenoids, total phenolic content (TPC), total antioxidant capacity (ABTS), and total anthocyanin content (TAC) of two lettuce cultivars grown in the second crop cycle under three photoperiods and with the same DLI.

	Chlorophylls	Carotenoids	TPC	ABTS	TAC
	mg·g^-1^	mg·g^-1^	mg GAE·g^-1^	µmol TE·g^-1^	mg Mv-3-glu·g^-1^
Photoperiod (P)					
16 L:8 D	5.64 ± 0.69	1.56 ± 0.10	29.02 ± 1.76 a	100 ± 49.71	5.41 ± 3.32 ab
12 L:12 D	5.42 ± 0.82	1.63 ± 0.38	26.91 ± 1.82 b	112 ± 74.88	6.01 ± 4.14 a
24 L:0 D	6.20 ± 0.99	1.60 ± 0.11	25.54 ± 1.62 b	98 ± 54.62	4.55 ± 2.78 b
Cultivar (Cv)					
Green	6.34 ± 0.48	1.46 ± 0.10	14.87 ± 1.43	61 ± 3.41	0.07 ± 0.03
Red	5.16 ± 0.33	1.74 ± 0.15	39.42 ± 2.22	145 ± 16.92	10.51 ± 1.41
Significance ^(1)^					
P	ns	ns	*	ns	*
Cv	**	**	***	***	***
P x Cv	ns	*	ns	ns	*

Mean ± SD values are reported.

^(1)^ Significance: ***, ** and * for p ≤ 0.001, p ≤ 0.01 and p ≤ 0.05, respectively; ns, not significant. Different letters indicate significant differences (p = 0.05) between means within the same column (Tukey HSD).

## 4 Discussion

The results of this study show that, under controlled-environment conditions and constant DLI, the three photoperiod/PPFD combinations tested: a) photoperiod 16:8 h (light/dark), with PPFD equal to 250 μmol·m⁻²·s⁻¹ (16 L:8 D); b) photoperiod 12:12 h (light/dark), with PPFD equal to 340 μmol·m⁻²·s⁻¹ (12 L:12 D); c) photoperiod 24:0 h (light/dark), with PPFD equal to 170 μmol·m⁻²·s⁻¹ (24 L:0 D), produced comparable outcomes in terms of lettuce morphology, yield, and photosynthetic performance (**Tables 1**, **2**). This finding contrasts with previous studies reporting growth stimulation under extended photoperiods or continuous lighting (Koontz and Prince, 1986; Chen et al., 2022; Miao et al., 2023). The absence of significance differences may be explained by the moderate PPFD levels used in our treatments, which likely prevented both photoinhibition under shorter photoperiods and physiological stress under continuous light, aligning with the concept that lettuce is highly adaptable to a wide range of light regimes when DLI is kept constant (Kelly et al., 2020; Pennisi et al., 2020).

Chlorophyll fluorescence parameters further confirm this stability (**Supplementary Tables S1**, **S2**). In fact, Y(II), NPQ, ETR, and Fv/Fm values fall within the expected physiological range for healthy lettuce plants (Hernández-Adasme et al., 2025), indicating that none of the photoperiods imposed measurable photochemical stress. Likewise, the light-response parameters (ETRmax, Alpha, Ik) remained unchanged among treatments, confirming that the intrinsic electron transport capacity was unaffected by photoperiod (Kaiser et al., 2024). Gas exchange responses were equally stable, across treatments (**Table 3**), supporting the notion that photosynthesis was operating under non-limiting light conditions (Zhang et al., 2018; Zhou et al., 2020; Jin et al., 2023).

Continuous light (24 L:0 D) has kept stable lettuce yield (**Table 2**) and reduced LED electricity costs, thereby improving energy use efficiency (EUE) and light use efficiency (LUE) compared with 12 L:12 D and 16 L:8 D photoperiods (**Table 4**). These improvements derive from distributing the same DLI over a longer photoperiod, which reduces instantaneous power demand and enhances lighting efficiency, as previously reported for VFs (Graamans et al., 2018; Stanghellini and Katzin, 2024). Nevertheless, artificial lighting remained the dominant energy cost, in line with reports indicating that lighting typically accounts for 60–85% of total energy consumption in indoor farming systems (Yorifuji and Obara, 2022).

Recently, it was shown that, in lettuce, variations in growth and nutritional traits are largely driven by changes in DLI, rather than by photoperiod alone, with similar plant responses when equivalent DLI levels are achieved through either increased PPFD or extended lighting duration (Min et al., 2026). Accordingly, the lack of significant differences in plant morphology, photosynthetic activity and chlorophyll fluorescence parameters observed in the present study can be attributed to the experimental maintenance of a constant DLI across photoperiod treatments, which likely ensured stable carbon assimilation and prevented photochemical stress. While most previous work has focused on plant-level responses under variable DLI conditions, our findings indicate that, when DLI is held constant, modifying the temporal distribution of light has limited effects on lettuce physiology but can substantially affect energy and cost performance of the VFs, thereby underpinning the improvements in energy use efficiency and light use efficiency observed under continuous low-intensity lighting.

In contrast to the lack of photoperiod effects, several cultivar-dependent differences emerged. Green lettuce produced more biomass, while red lettuce exhibited higher concentrations of carotenoids, phenolic compounds, and antioxidant activity, patterns consistent with intrinsic pigment composition and secondary metabolism of red-leaf cultivars (DuPont et al., 2000; Stratil et al., 2006; Liu et al., 2007; Llorach et al., 2008; Cruz et al., 2012). The particularly high anthocyanin and carotenoid levels observed in red lettuce under 12 L:12 D (**Table 5**) align with previous studies suggesting that the presence of a dark period may promote the synthesis or stabilization of certain photoprotective compounds (Caldwell et al., 2003; Kim et al., 2018; Zhao et al., 2007; Rao and Rao, 2007). However, since photoperiod did not influence overall growth or photochemistry, these metabolic adjustments appear decoupled from biomass accumulation, supporting the view that pigment biosynthesis and resource allocation follow genotype-specific regulatory pathways (Ouzounis et al., 2015; Yang et al., 2022).

The consistency of morphological, physiological, and biochemical responses across two experimental cycles highlights the robustness of controlled-environment systems, where environmental variability is minimized and plant responses are highly reproducible (van Delden et al., 2021; Gruda et al., 2025). Nevertheless, some limitations should be considered. The study tested only two crisphead cultivars, and responses may differ in other lettuce types or genotypes with distinct photobiological traits (Modarelli et al., 2022). Additionally, because DLI was maintained constant across treatments, the results cannot be generalized to scenarios where photoperiod and DLI vary simultaneously, as commonly occurs in commercial VFs (Arcasi et al., 2024; Kaiser et al., 2024). Finally, the economic evaluation was based on specific energy tariffs and may vary depending on geographic and temporal factors.

Despite these limitations, the findings provide valuable insights into light management in VFs. When the DLI is kept constant, photoperiod adjustments may have limited impact on growth and photosynthesis but can significantly affect energy efficiency.

## 5 Conclusions

This study clarifies that, under controlled conditions and with a fixed DLI, lettuce can sustain stable growth and photosynthetic performance across a wide range of photoperiod–PPFD combinations. This finding delineates a functional flexibility in light scheduling that can be exploited to optimize EUE without compromising crop performance. In particular, the evidence that continuous lighting improves EUE while maintaining morphological and physiological stability provides a concrete rationale for adopting extended photoperiods in cost-sensitive VFs scenarios.

The work also underscores the central role of genotype in shaping nutritional and pigment profiles, indicating that cultivar choice may influence market-oriented quality attributes more strongly than lighting duration when the daily light integral is kept constant. Nevertheless, it is well established that quality-related traits can be further modulated through additional abiotic management strategies, including root-zone temperature control, targeted or dynamic light spectrum modulation (e.g., end-of-cycle blue light enrichment), and other controlled-environment stressors, which can partially enhance or steer nutritional and pigment accumulation independently of genotype.

Looking forward, these results support the development of next-generation lighting strategies that move beyond fixed photoperiods toward dynamic, plant-responsive, and energy-aware lighting management. Future studies should integrate dynamic lighting strategies, plant-feedback systems, and spectral optimization to achieve a more holistic improvement in sustainability and productivity. Greater attention should also be given to genotype-specific light requirements, particularly regarding secondary metabolism and nutritional quality, to refine cultivar selection in controlled-environment.

## Data Availability

The raw data supporting the conclusions of this article will be made available by the authors, without undue reservation.
