# Supplementary material for: Continuous lighting at low PPFD improves energy efficiency while preserving growth and quality of lettuce in vertical farming systems
Source: Front Plant Sci. 2026 Mar 4;17:1783548. doi: 10.3389/fpls.2026.1783548 (PMC12995675; doi:10.3389/fpls.2026.1783548)
Supplement: Supplementary file 1 [file Table1.docx]

Table S1. Effective quantum yield of PSII (Y(II)), non-photochemical quenching (NPQ), electron transport rate (ETR) and the maximum quantum efficiency of PSII (Fv/Fm) of two lettuce cultivars, 'Falstaff' (green) and 'Copacabana' (red), grown under three photoperiods and with the same DLI. Mean ± SE values are reported.

|  | Y(II) | NPQ | ETR | Fv/Fm |
| --- | --- | --- | --- | --- |
|  | µmol·m^-2^ s^-1^ | | | |
| Photoperiod (P) |  |  |  |  |
| 16 L:8 D | 0.44 ± 0.03  3 | 1.02 ± 0.11 | 51.1 ± 3.2 | 0.79 ± 0.01 |
| 12 L:12 D | 0.39 ± 0.04 | 1.13 ± 0.17 | 55.7 ± 5.1 | 0.78 ± 0.01 |
| 24 L:0 D | 0.52 ± 0.02 | 0.71 ± 0.11 | 43.0 ± 2.0 | 0.78 ± 0.02 |
| Cultivar (Cv) |  |  |  |  |
| Green | 0.44 ± 0.05 | 0.92 ± 0.20 | 48.9 ± 5.4 | 0.78 ± 0.02 |
| Red | 0.46 ± 0.05 | 0.98 ± 0.21 | 51.1 ± 6.2 | 0.79 ± 0.02 |
|  |  |  |  |  |
| Significance^(1)^ |  |  |  |  |
| P | ns | ns | ns | ns |
| cv | ns | ns | ns | ns |
| P x Cv | ns | ns | ns | ns |

^(1)^ Significance: ns, not significant.
