# Supplementary material for: Continuous lighting at low PPFD improves energy efficiency while preserving growth and quality of lettuce in vertical farming systems
Source: Front Plant Sci. 2026 Mar 4;17:1783548. doi: 10.3389/fpls.2026.1783548 (PMC12995675; doi:10.3389/fpls.2026.1783548)
Supplement: Supplementary file 2 [file Table2.docx]

Table S2. Maximum quantum yield for whole chain electron transport at low light intensities (*Alpha*), maximum electron transport capacity at light saturation (*ETRmax*), and light saturation coefficient (*Ik*) of two lettuce cultivars, 'Falstaff' (green) and 'Copacabana' (red), grown under three photoperiods and with the same DLI. Mean ± SE values are reported.

|  | ETR max | Alpha | Ik |
| --- | --- | --- | --- |
|  | µmol·m^-2^ s^-1^ | µmol photons·m^-2^·s^-1^ | |
| Photoperiod (P) |  |  |  |
| 16 L:8 D | 62.1 ± 11.8 | 0.39 ± 0.05 | 167.5 ± 37.3 |
| 12 L:12 D | 66.6 ± 10.0 | 0.37 ± 0.02 | 181.5 ± 27.9 |
| 24 L:0 D | 58.4 ± 10.9 | 0.37 ± 0.02 | 162.5 ± 33.6 |
| Cultivar (Cv) |  |  |  |
| Green | 55.7 ± 10.9 | 0.37 ± 0.05 | 161.2 ± 38.4 |
| Red | 69.0 ± 14.2 | 0.39 ± 0.02 | 179.5 ± 42.2 |
| Significance^(1)^ |  |  |  |
| P | ns | ns | ns |
| Cv | ns | ns | ns |
| P x Cv | ns | ns | ns |

^(1)^ Significance: ns, not significant.
