# Supplementary material for: Continuous lighting at low PPFD improves energy efficiency while preserving growth and quality of lettuce in vertical farming systems
Source: Front Plant Sci. 2026 Mar 4;17:1783548. doi: 10.3389/fpls.2026.1783548 (PMC12995675; doi:10.3389/fpls.2026.1783548)
Supplement: Supplementary file 3 [file Table3.docx]

**Table S3. Colorimetric parameters of green lettuce cultivar, 'Falstaff', grown under three photoperiods and with the same DLI. Mean ± SE values are reported.**

|  | aD65* | bD65* | L*D65* | Chroma |
| --- | --- | --- | --- | --- |
| Photoperiod |  |  |  |  |
| 16 L:8 D | -13.5 ± 0.54 | 19.8 ± 1.41 | 42.3 ± 1.50 | 24.0 ± 1.46 |
| 12 L:12 D | -13.4 ± 0.19 | 19.3 ± 0.64 | 42.4 ± 0.82 | 23.5 ± 0.60 |
| 24 L:0 D | -13.1 ± 0.49 | 18.9 ± 1.07 | 41.5 ± 0.98 | 22.9 ± 1.16 |
|  |  |  |  |  |
| Significance^(1)^ | ns | ns | ns | ns |

^(1)^ Significance: ns, not significant.

**Table S4. Colorimetric parameters of red lettuce cultivar, 'Copacabana', grown under three photoperiods and with the same DLI. Mean ± SE values are reported.**

|  | aD65* | bD65* | L*D65* | Chroma |
| --- | --- | --- | --- | --- |
| Photoperiod (P) |  |  |  |  |
| 16 L:8 D | 2.8 ± 0.52 | 0.7 ± 0.61 | 28.0 ± 0.47 | 3.1 ± 0.48 |
| 12 L:12 D | 2.1 ± 0.53 | 0.5 ± 0.26 | 28.6 ± 0.51 | 2.2 ± 0.51 |
| 24 L:0 D | 1.8 ± 0.88 | 1.7 ± 1.84 | 28.8 ± 1.29 | 3.6 ± 1.29 |
| Significance ^(1)^ |  |  |  |  |
| P | ns | ns | ns | ns |

^(1)^ Significance: ns, not significant.
